# RNF126 Quenches RNF168 Function in the DNA Damage Response

**DOI:** 10.1016/j.gpb.2018.07.004

**Published:** 2018-12-04

**Authors:** Lianzhong Zhang, Zhenzhen Wang, Ruifeng Shi, Xuefei Zhu, Jiahui Zhou, Bin Peng, Xingzhi Xu

**Affiliations:** 1 College of Life Sciences, Capital Normal University, Beijing 100048, China; 2 Faculty of Life Sciences, Tangshan Normal College, Tangshan 063000, China; 3 Guangdong Key Laboratory for Genome Stability & Disease Prevention, Shenzhen University Health Science Center, Shenzhen 518060, China

**Keywords:** DNA repair, DNA damage response, Ubiquitination, RNF126, RNF8, RNF168

## Abstract

**DNA damage response** (DDR) is essential for maintaining genome stability and protecting cells from tumorigenesis. Ubiquitin and ubiquitin-like modifications play an important role in DDR, from signaling DNA damage to mediating **DNA repair**. In this report, we found that the E3 ligase ring finger protein 126 (**RNF126**) was recruited to UV laser micro-irradiation-induced stripes in a **RNF8**-dependent manner. RNF126 directly interacted with and ubiquitinated another E3 ligase, **RNF168**. Overexpression of wild type RNF126, but not catalytically-inactive mutant RNF126 (CC229/232AA), diminished **ubiquitination** of H2A histone family member X (H2AX), and subsequent bleomycin-induced focus formation of total ubiquitin FK2, TP53-binding protein 1 (53BP1), and receptor-associated protein 80 (RAP80). Interestingly, both RNF126 overexpression and RNF126 downregulation compromised homologous recombination (HR)-mediated repair of DNA double-strand breaks (DSBs). Taken together, our findings demonstrate that RNF126 negatively regulates RNF168 function in DDR and its appropriate cellular expression levels are essential for HR-mediated DSB repair.

## Introduction

Genomic DNA is under continuous assault by various environmental and endogenous factors that cause DNA damage, including UV radiation from sunlight and free radicals derived from intermediate metabolites. As such, a comprehensive DNA damage response (DDR) network has evolved that can repair damaged DNA, maintain genome stability, and protect cells from tumorigenesis [1], [2], [3]. Deficiencies in the DDR lead to accumulation of DNA damage and ultimately genome instability and cancer [1]. However, deliberate induction of DNA damage via radiation therapy or exposure to certain chemotherapeutic agents is a valuable strategy to cause irreparable DNA damage and promote cancer cell death [4], [5]. Therefore, fully understanding the mechanisms that underlie the DDR network will help delineate the molecular mechanisms underlying tumorigenesis, determine measures of cancer prevention, and develop novel and effective cancer therapies.

DNA double-strand breaks (DSBs) are the most deleterious form of DNA damage, because one unrepaired DSB is sufficient to induce cell death [6]. DSBs are repaired by either the high-fidelity process of homologous recombination (HR), or the error-prone processes of non-homologous end-joining (NHEJ) or microhomology-mediated end joining (MMEJ) [1]. HR-mediated repair depends on the presence of a sister chromatid and thus must occur in G2/S phase [7]. NHEJ-mediated repair occurs throughout the cell cycle, although mainly in the G1 phase [8], and MMEJ, serving as an alternative to NHEJ, seals a subset of DSBs [9].

With the occurrence of DSBs, the Mre11/Rad50/Nbs1 (MRN) complex is among the first arrays of proteins to sense DNA damage and promote activation of ataxia telangiectasia mutated (ATM), the master protein kinase for DSB signaling [1], [2], [3]. Activated ATM in turn phosphorylates hundreds of substrates to mobilize and coordinate all the necessary cellular activities, ensuring DSB signaling and repair. For instance, serine 139 (S139) of histone variant H2A histone family member X (H2AX) is phosphorylated by ATM [10]. This phosphorylation on the chromatin regions surrounding a DSB serves as a platform for recruitment and enrichment of moderators and repair proteins [11], [12]. ATM-mediated phosphorylation of mediator of DNA damage checkpoint protein 1 (MDC1) promotes its oligomerization [13] and initiates a switch of DSB signaling from being extensively driven by phosphorylation to ubiquitination through recruiting pivotal E3 ligases including ring finger protein 8 (RNF8) and RNF168 to the DSB sites [14], [15], [16], triggering ubiquitination of many proteins, including H2A/H2AX [17], [18], and subsequent enrichment of DDR factors, including TP53-binding protein 1 (53BP1), breast cancer type 1 susceptibility protein (BRCA1), receptor-associated protein 80 (RAP80), to facilitate DSB repair.

Ubiquitination is carried out by a cascade of enzymatic reactions involving ubiquitin-activating enzymes (E1s), ubiquitin-conjugating enzymes (E2s), and ubiquitin ligases (E3s) that control substrate specificity [19]. RNFs are a group of >300 proteins containing a really interesting new gene (RING) structural domain characteristic of a C3HC4-type zinc finger [20], [21]. Many RNFs, like RNF8 and RNF168, have an important role in the DDR and are tightly modulated by other RNFs (*e.g.*, RNF169 [22], [23], [24]) and scaffold proteins, *e.g.*, histone H1 [18], B-cell lymphoma/leukemia 10 (BCL10) [25], and lethal (3) malignant brain tumor-like 2 (L3MBTL2) [26]).

The reversal of ubiquitination is achieved by deubiquitinating enzymes (DUBs) that specifically disassemble ubiquitin chains and maintain a balance between ubiquitination and deubiquitination [27]. The human genome encodes ∼600 E3 ligases and ∼100 DUBs; >20 E3 ligases and >12 DUBs reportedly modulate the DDR [28], [29]. Our lab has long been interested in identifying and characterizing novel E3 ligases and DUBs involved in the DDR and how they interact with RNF8/RNF168 [25], [30]. In this study, we found that RNF126 is recruited to DSB sites in a RNF8-dependent manner, where it negatively regulates RNF168-mediated ubiquitination of H2AX and recruitment of DDR factors downstream of RNF168. We further demonstrated that maintaining proper levels of RNF126 is essential for HR-mediated DSB repair.

## Results

### RNF126 accumulates at DSBs independent of its catalytic activity

**Figure 1:**
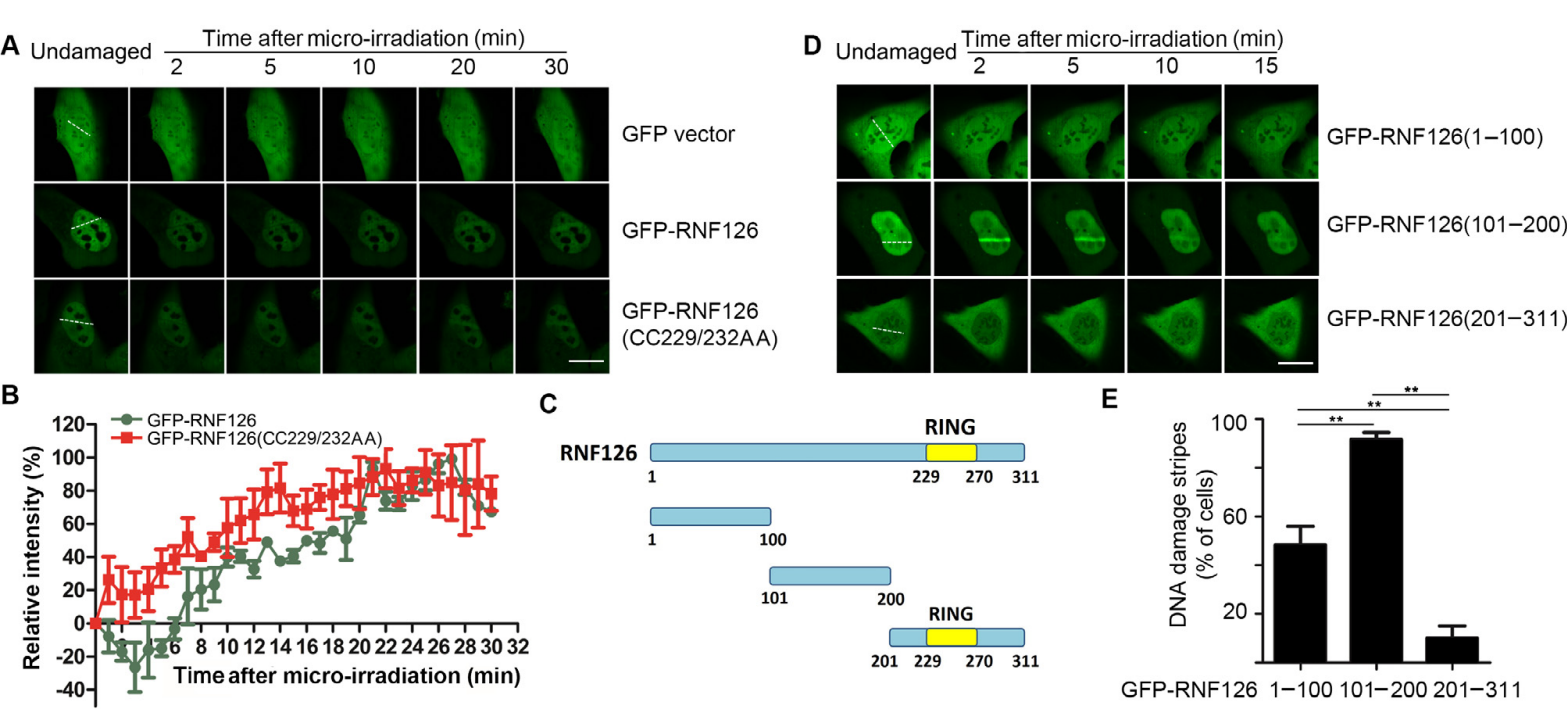
**RNF126 is recruited to the UV laser micro-irradiation-induced DNA damage stripes** **A.** and **B.** Both wild-type (WT) RNF126 and catalytically-inactive RNF126(CC229/232AA) are recruited to DNA damage stripes. U2OS cells transiently expressing GFP-RNF126 or GFP-RNF126(CC229/232AA) were irradiated with a 365-nm UV laser beam (white dashed line). Images were collected every 30 sec after irradiation and representative images are shown (A). The recruitment kinetics of GFP-RNF126 (WT) and GFP-RNF126(CC229/232AA) were assessed in terms of signal intensity at DNA damage stripes relative to the un-irradiated area in three independent experiments (B). Data represent the mean ± SD. **C.** Diagram depicting the domain structure of RNF126 and its truncation mutants containing the N-terminus (amino acid residues 1–100), middle region (amino acid residues 101–200), and C-terminus (amino acid residues 201–311), respectively. **D.** and **E.** U2OS cells expressing GFP-tagged RNF126 truncation mutants were subjected to UV laser micro-irradiation and the recruitment of GFP fusion proteins to the DNA damage stripes were monitored in live cells. Representative images are shown (D). The percentage of cells positive for GFP fusion protein enrichment at DNA damage stripes was determined by analyzing >100 GFP-positive cells for each GFP fusion protein (E). Data represent the mean ± SD. A two-way ANOVA was performed. ^**^P < 0.01. Scale bar, 10 μm. RING, really interesting new gene; RNF, ring finger protein.

**Supplementary Figure S1:**
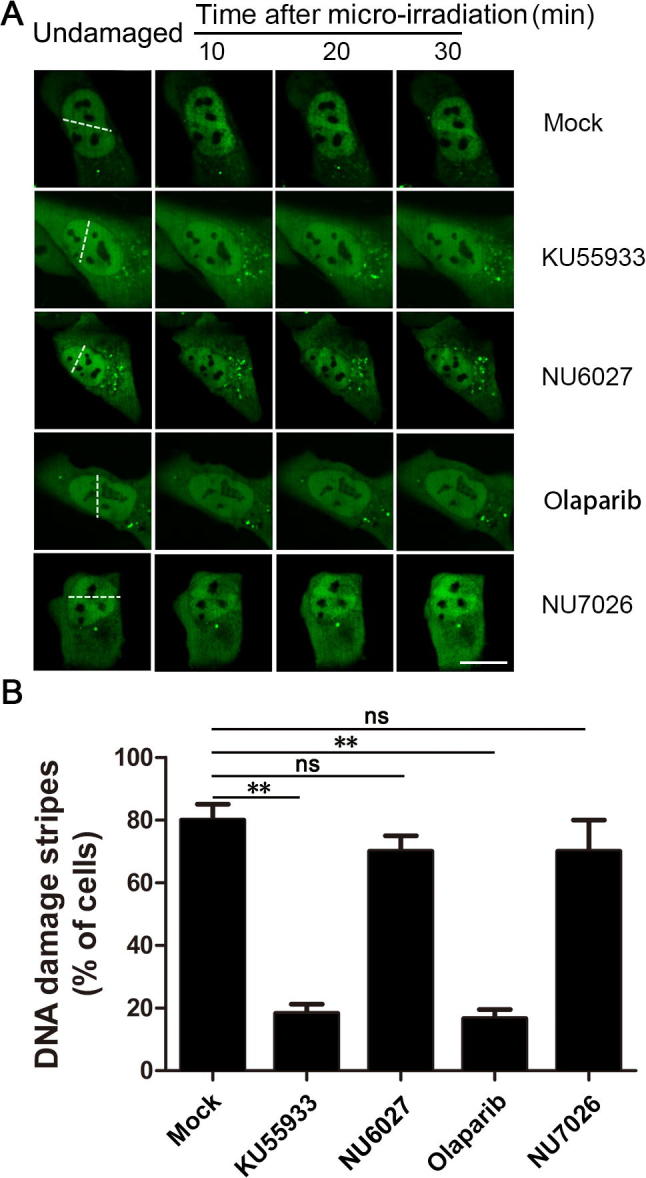
**RNF126 recruitment to the DNA damage stripe is dependent on PARP1 and ATM A.** U2OS cells transiently expressing GFP-RNF126 were pre-treated with mock control (DMSO), ATM inhibitor KU55933, ATR inhibitor NU6027, PARP inhibitor olaparib, or DNA-PKcs inhibitor NU7026 for 1 h before subjected to micro-irradiation with a 365-nm UV laser beam (white dashed line). Images were collected every 30 s after irradiation and representative images are shown. **B.** The percentage of cells positive for GFP-RNF126 enrichment at DNA damage stripes was determined by analyzing >100 GFP-positive cells for each treatment. Data represent the mean ± SD. A two-way ANOVA was performed. **P < 0.01. ns, not significant. Scale bar, 10 μm. ATM, ataxia telangiectasia mutated; ATR, ataxia telangiectasia and Rad3-related protein; DNA-PKcs, DNA-dependent protein kinase catalytic subunit; PARP1, poly [ADP-ribose] polymerase 1.

A previous study that used a ubiquitin-activated interaction trap identified RAD50 as a potential RNF126 substrate [31]. This finding led us to speculate that RNF126 could be involved in the DDR. To test this possibility, we over-expressed GFP-tagged wild-type RNF126 or catalytically-inactive mutant RNF126(CC229/232AA) in U2OS cells and monitored the spatiotemporal accumulation of GFP at micro-irradiated stripes generated with a 365-nm UV laser beam. GFP-RNF126 dispersed from the irradiated stripes during the first several minutes after micro-irradiation, but then re-accumulated 10 min after micro-irradiation and persisted at least for 30 min (Figure 1A and B). Unexpectedly, the catalytically-inactive mutant GFP-RNF126(CC229/232AA) exhibited similar recruitment dynamics at the DNA damage stripes (Figure 1A and B), suggesting that RNF126 recruitment to sites of DNA damage is independent of its catalytic activity. To determine if RNF126 recruitment to the DNA damage stripe is dependent on the catalytic activity of ATM, ataxia telangiectasia and Rad3-related protein (ATR), DNA-dependent protein kinase catalytic subunit (DNA-PKcs), or poly [ADP-ribose] polymerase 1 (PARP1), U2OS cells expressing GFP-RNF126 was pre-treated with specific inhibitor respectively. We found that both ATM inhibitor and PARP inhibitor efficiently diminished recruitment of GFP-RNF126 to the DNA damage stripes, whereas neither ATR inhibitor nor DNA-PKcs inhibitor had such an effect (Figure S1), suggesting that RNF126 recruitment to the DNA damage site is dependent on ATM and PARP.

The N-terminus of RNF126 [amino acid residues (aa) 1–100] has been shown to be required for its interaction with BCL2-associated athanogene 6 (BAG6) [32], while its C-terminus (aa 201–311) contains a RING domain that is required for its interaction with activation-induced cytidine deaminase (AID) [33]. We thus mapped the RNF126 domain(s) required for its localization to DNA damage sites, using three truncated GFP-tagged RNF126 fragments spanning the full-length polypeptide, that is, 1–100, 101–200, and 201–311 (Figure 1C). Here, we found that GFP-RNF126(101–200) accumulated at the DNA damage stripe as early as 2 min after irradiation in the majority (90%) of transfected U2OS cells, compared to only 50% of GFP-RNF126(1–100) transfected cells. However, we did not detect any GFP-RNF126(201–311) accumulation at the DNA damage stripe (Figure 1D and E). These results indicate that the middle region of the RNF126 polypeptide (aa 101–200) is predominantly responsible for mediating its accumulation at DNA damage sites.

### RNF126 interacts with RNF8 and RNF168

**Figure 2:**
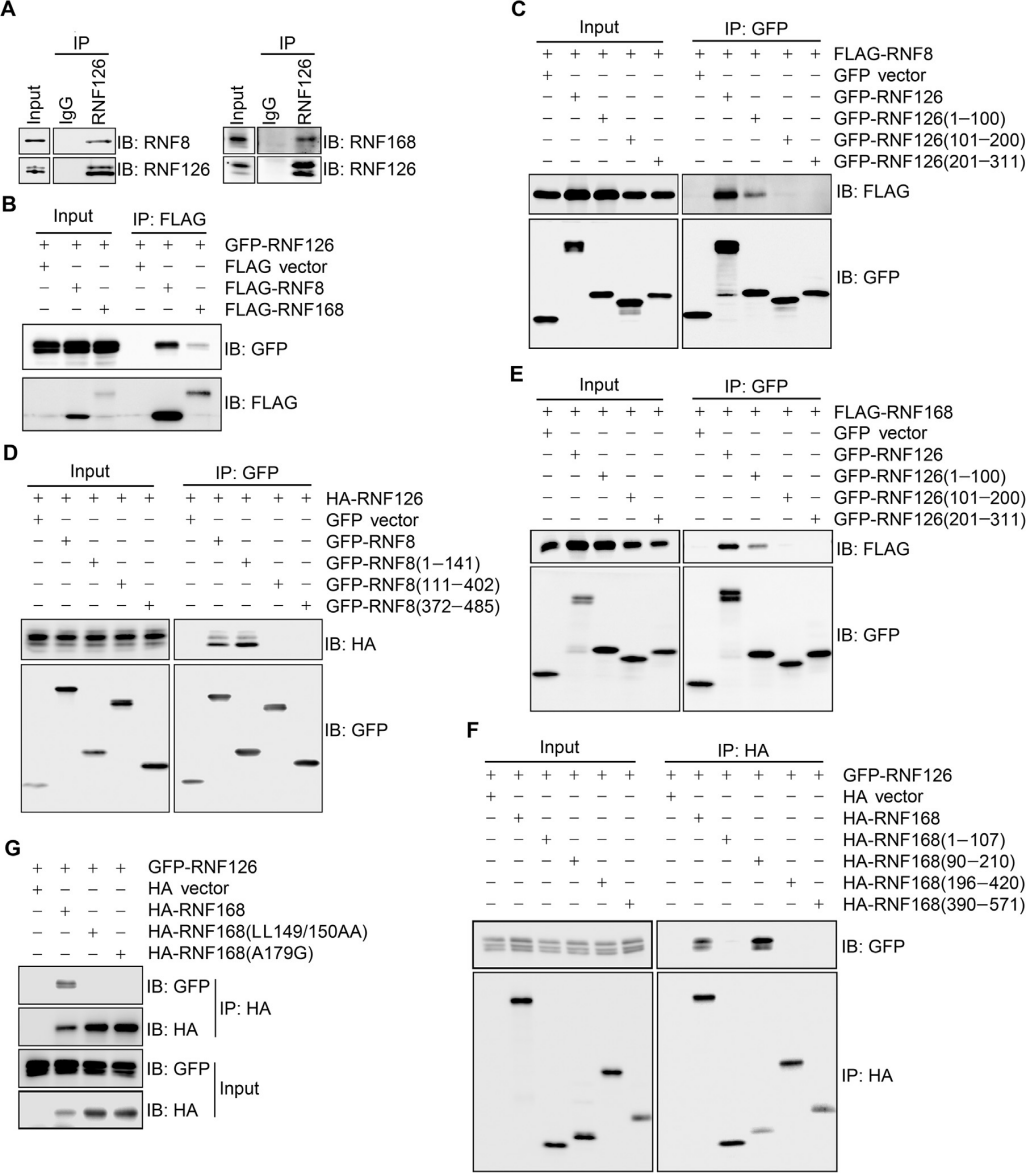
**RNF126 interacts with RNF8 and RNF168** **A.** Endogenous RNF126 interacts with both RNF8 and RNF168. Total lysates from HEK293T cells were immunoprecipitated with an anti-RNF126 antibody, and the immunocomplexes were exposed to the indicated antibodies. **B.** Epitope-tagged RNF126 interacts with RNF8 and RNF168. A GFP-RNF126 expression construct was co-transfected with FLAG-RNF8 or FLAG-RNF168 in HEK293T cells. Total lysates were harvested 48 h after transfection and subjected to immunoprecipitation with anti-FLAG beads followed by immunoblotting with the indicated antibodies. **C.** The RNF126 N-terminus (amino acid residues 1–100) interacts with RNF8. FLAG-RNF8 was co-expressed with GFP-RNF126 or its truncation mutants in HEK293T cells. **D.** The FHA domain-containing N-terminus of RNF8 (amino acid residues 1–141) interacts with RNF126. HA-RNF126 was co-expressed with GFP-RNF8 or its truncation mutants in 293T cells. **E.** The RNF126 N-terminus interacts with RNF168. FLAG-RNF168 was co-expressed with GFP-RNF126 or its truncation mutants in HEK293T cells. **F.** The UMI and MIU1 domains-containing region of RNF168 (amino acid residues 90–210) interact with RNF126. GFP-RNF126 was co-expressed with HA-RNF168 or its truncation mutants in HEK293T cells. **G.** Both UMI domain and MIU1 domain of RNF168 are essential for its interaction with RNF126. GFP-RNF126 was co-expressed with wild-type HA-RNF168, the UMI point mutant HA-RNF168(LL149/150AA), or the MIU1 point mutant HA-RNF168(A179G) in HEK293T cells. In panels C–G, total cell lysates were harvested 48 h after transfection and subjected to immunoprecipitation and immunoblotting with the indicated antibodies. IB, immunoblot; IP, immunoprecipitation; FHA, forkhead-associated; MIU1, motif interacting with ubiquitin 1; UMI, ubiquitin interacting motif and MIU-related ubiquitin binding domain.

We next wanted to determine whether RNF126 interacts with RNF8 or RFN168, the two critical E3 ligases required during the DDR [1]. By co-immunoprecipitation, we found that both endogenous and FLAG-tagged RNF8 and RNF168 immunoprecipitated with RNF126 or GFP-RNF126 in HEK293T cells (Figure 2A and B). These data demonstrate that RNF126 physically interacts with RNF8 and RNF168.

**Supplementary Figure S2:**
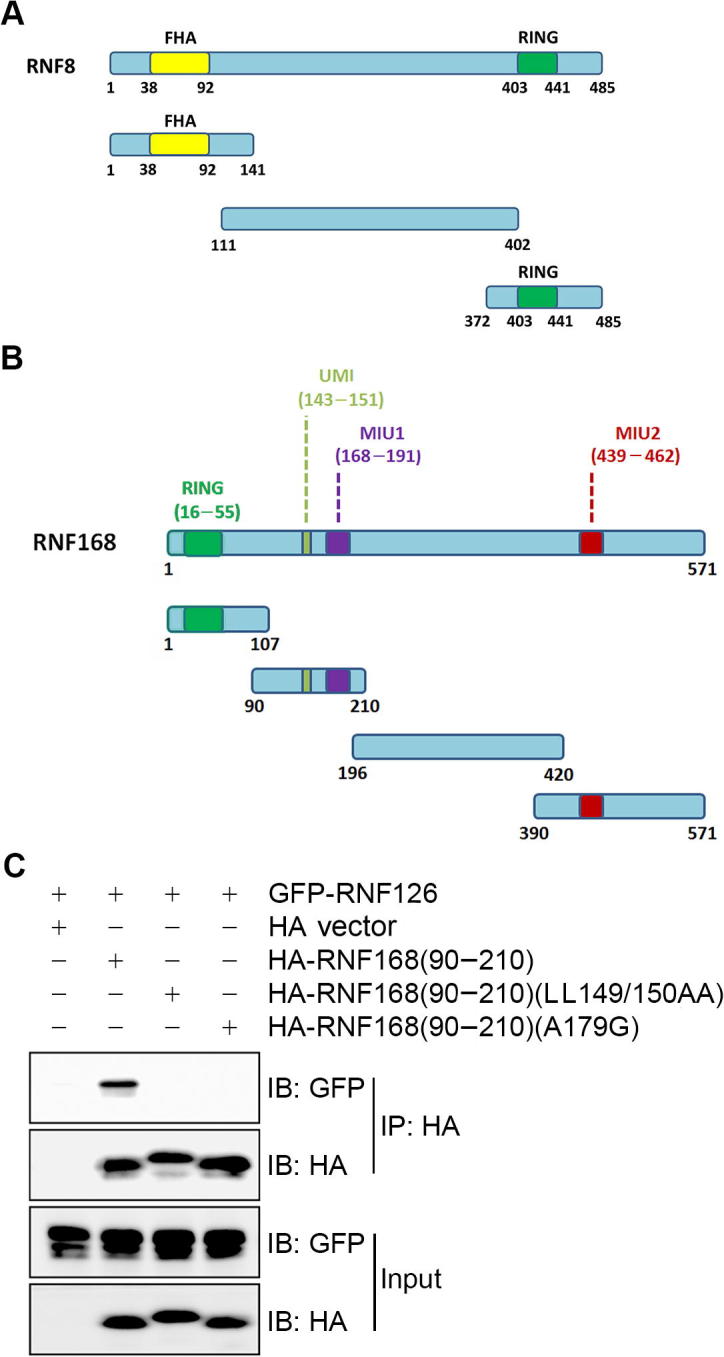
**RNF126 interacts with RNF8 and RNF168 A.** Diagram depicting the domain structure of RNF8 and its truncation mutants. **B.** Diagram depicting the domain structure of RNF168 and truncation mutants. **C.** Both the UMI and MIU1 domains of RNF168 are essential to mediate the interaction between RNF168 and RNF126. GFP-RNF126 was co-expressed with wild-type HA-RNF168(90−210), the UMI point mutant HA-RNF168(90−210)(LL149/150AA), or the MIU1 point mutant HA-RNF168(90−210)(A179G) in HEK293T cells. Total cell lysates were harvested 48 h after transfection and subjected to immunoprecipitation and immunoblotting with the indicated antibodies. IB, immunoblot; FHA, forkhead-associated; MIU1, motif interacting with ubiquitin; UMI, ubiquitin interacting motif and MIU-related ubiquitin binding domain.

We then mapped the binding domains mediating these interactions between RNF126 and RNF8/RNF168 by co-transfecting the truncated GFP-tagged RNF126 fragments with FLAG-tagged RNF8 or HA-RNF168 in HEK293T cells. Here, FLAG-tagged RNF8 immunoprecipitated with the GFP-tagged RNF126 N-terminus (aa 1–100), but not its middle region (aa 101–200), or C-terminus (aa 201–311) (Figure 2C). Reciprocally, the N-terminus of RNF8(aa 1–141), which contains a forkhead-associated domain (FHA), but not the RING domain or the middle region of RNF8, physically interacted with full length RNF126 (Figures S2A and 2D). It is noted that both RNF8 and RNF168 bind to the N terminus of RNF126, indicating that RNF8 and RNF168 may sequentially bind to RNF126 during DDR given that RNF168 enrichment at DSBs is RNF8-dependent, while the recruitment of RNF126 to the DNA damage site predominantly through its middle region would allow its flexible N terminus to dynamically interact with different partners.

The GFP-tagged RNF126 N terminus (aa 1–100), but not the middle region (aa 101–200) or the C terminus (aa 201–311), also co-immunoprecipitated with full-length HA-RNF168 (Figures S2B and 2E). On the other hand, full-length HA-RNF168 (Figure 2G) co-immunoprecipitated with GFP-RNF126 in HEK293T cells. The HA-RNF168 N-terminal fragment (aa 90–210), which contains motif interacting with ubiquitin 1 (MIU1) domain and ubiquitin interacting motif and MIU-related ubiquitin binding domain (UMI). This N-terminal HA-RNF168 fragment interacted with full-length GFP-RNF126 (Figure 2F), whereas mutations in these domains, UMI (LL149/150AA) and MIU1 (A179G), in the context of HA-RNF168(90–210) (Figure S2C) or full-length HA-RNF168 (Figure 2G) prevented the interaction with full-length GFP-RNF126. Taken together, these mapping results demonstrate that the N-terminus of RNF126 binds to the N-terminal FHA domain of RNF8 and the N-terminal UMI and MIU1 domains of RNF168.

### RNF126 recruitment to DSBs is RNF8-dependent

**Figure 3:**
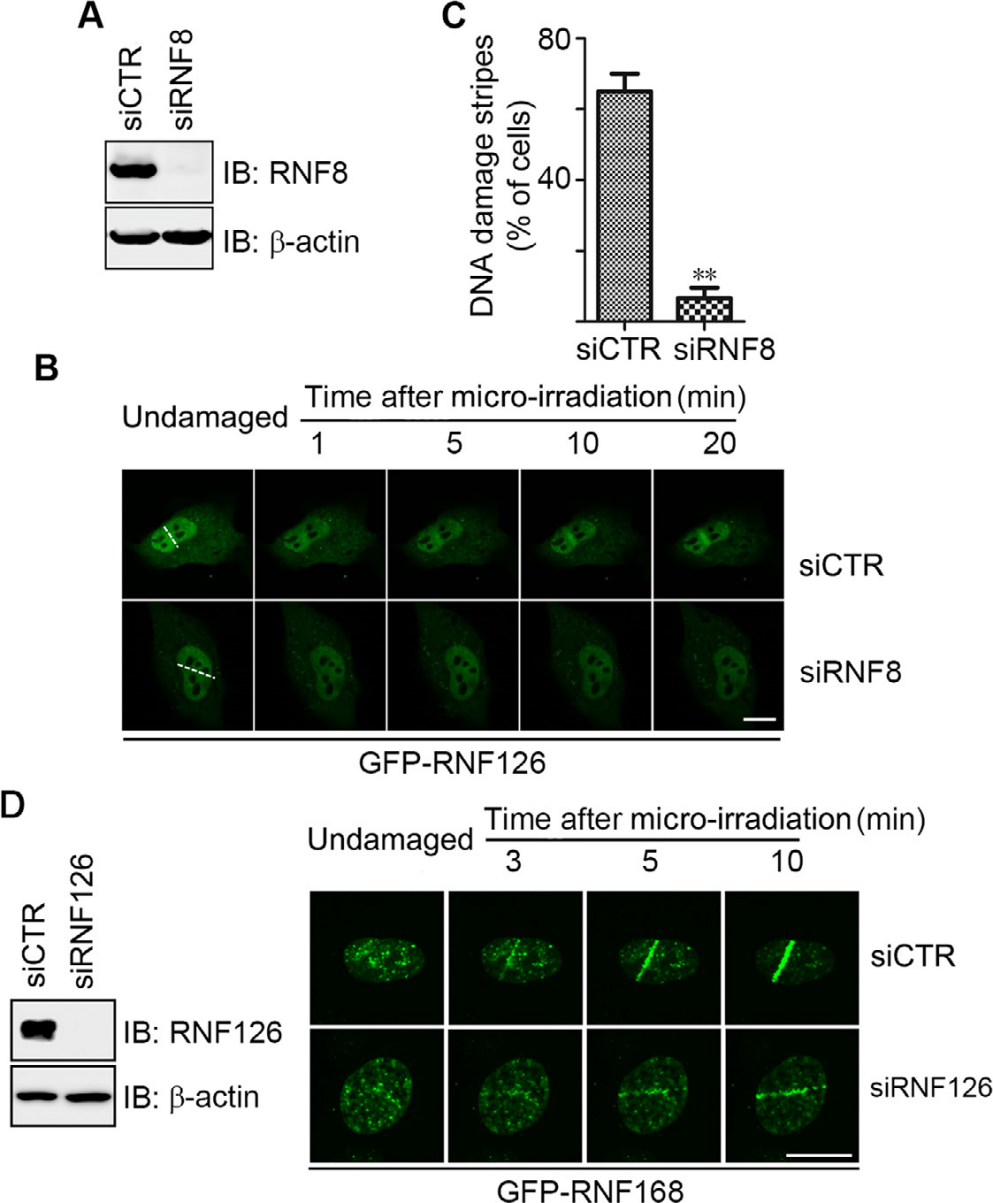
**RNF126 recruitment to DNA damage sites is RNF8-dependent** **A.** The siRNF8 knockdown efficiency was determined by immunoblotting using the indicated antibodies. **B.** Representative images of GFP-RNF126 recruitment to DNA damage stripes (white dashed line). **C.** Percentage of cells exhibiting GFP-RNF126 recruitment to DNA damage stripes, quantitated from three independent experiments, for each of which at least 100 GFP-positive cells were evaluated. Data represent the mean ± SD. A two-way ANOVA was performed. ^**^P < 0.01. In panels A–C, U2OS cells expressing GFP-RNF126 were transfected with a control siRNA oligo (siCTR) or a siRNA oligo specifically targeting RNF8 [siRNF8 (3′UTR)-1)]. **D.** Inhibition of RNF126 expression has no obvious impact on RNF168 recruitment to DNA damage sites. U2OS cells expressing GFP-RNF168 were transfected with a control siRNA oligo (siCTR) or a siRNA oligo specifically targeting RNF126 (siRNF126-A) and subjected to UV laser micro-irradiation 48 h after transfection. Representative images of GFP-RNF168 recruitment to DNA damage stripes are shown. IB, immunoblot. Scale bar, 10 μm.

**Supplementary Figure S3:**
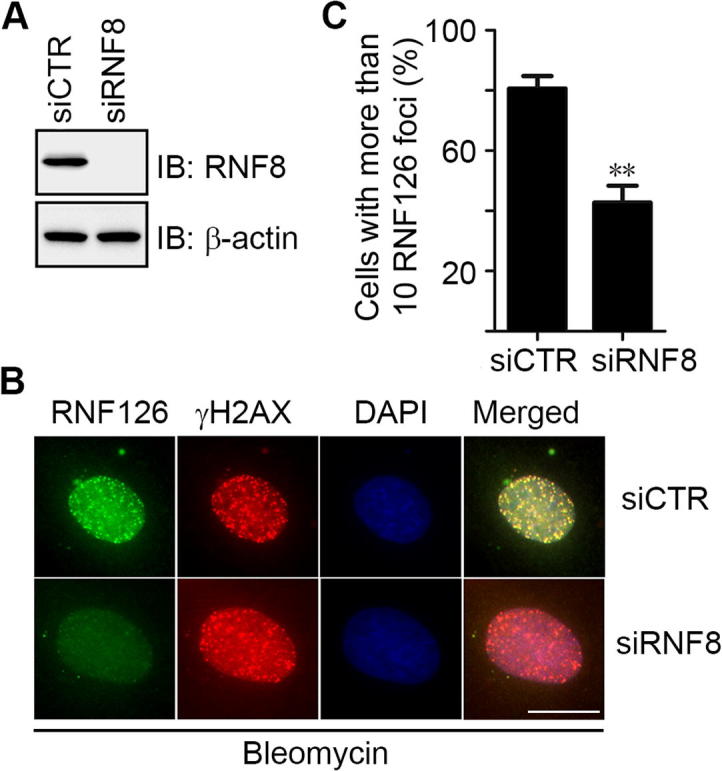
**RNF126 recruitment to DNA damage sites is RNF8-dependent** U2OS cells were transfected with a control siRNA oligo (siCTR) or a siRNA oligo specifically targeting RNF8 [siRNF8(3’UTR-1)]. **A.** The siRNF8 knockdown efficiency was determined by immunoblotting with the indicated antibodies. **B.** Representative images of DNA damage-induced RNF126 foci and γH2AX. The transfected cells were treated with bleomycin (10 mg/ml) for 1 h before co-immunofluorescence staining with anti-RNF126 (green) and anti-γH2AX (red). Genomic DNA was stained with DAPI (blue). **C.** The percentage of cells with >10 RNF126 foci was quantitated from three randomly selected fields on the condition that each field had >100 cells. Data represent the mean ± SD. A two-way ANOVA was performed. **P < 0.01. IB, immunoblot. Scale bar, 10 μm.

We next investigated whether RNF126 recruitment to DSBs is dependent on RNF8. Here, we used siRNA to inhibit RNF8 expression in U2OS cells (Figure 3A) and then monitored the recruitment dynamics of GFP-RNF126 to DSBs before and after UV micro-irradiation. GFP-RNF126 recruitment to DSBs was compromised in RNF8 siRNA knockdown cells (Figure 3B and C). This effect was confirmed by impaired formation of bleomycin-induced RNF126 foci following *RNF8* siRNA knockdown in U2OS cells (Figure S3). These data support that RNF126 enrichment at DSB sites occurs in a RNF8-dependent manner.

**Supplementary Figure S4:**
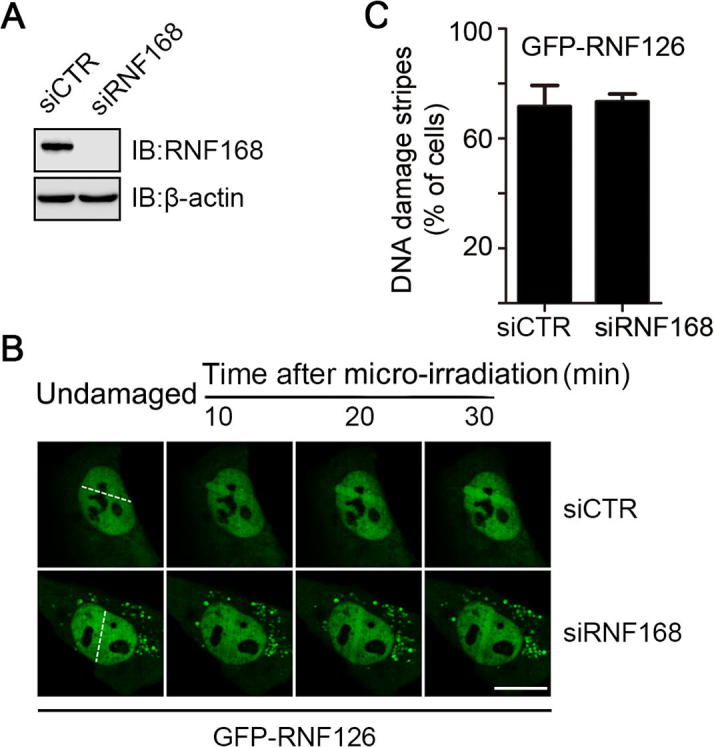
**RNF126 recruitment to DNA damage sites is RNF168-dependent** U2OS cells stably expressing GFP-RNF126 were transfected with a control siRNA oligo (siCTR) or a siRNA oligo specifically targeting RNF168 (siRNF168-A). **A.** The siRNF168 knockdown efficiency was determined by immunoblotting with the indicated antibodies. **B.** Representative images of UV laser micro-irradiation-induced RNF126 stripes were shown. **C.** The percentage of cells positive for GFP-RNF126 enrichment at DNA damage stripes was determined by analyzing >100 GFP-positive cells for each treatment. Data represent the mean ± SD. Scale bar, 10 μm.

We then dissected the interplay between RNF126 and RNF168 using siRNAs to inhibit the expression of RNF126 (Figure 3D) or RNF168 (Figure S4) in U2OS cells, and monitoring the recruitment of GFP-RNF168 or GFP-RNF126 to DNA damage stripes, respectively, before and after UV laser micro-irradiation. Inhibiting RNF126 expression did not markedly compromise RNF168 recruitment to DSB sites (Figure 3D). Neither down-regulation of RNF168 expression had an impact on RNF126 recruitment to DSB sites (Figure S4).

### RNF126-mediated RNF168 ubiquitination negatively regulates RNF168 function

**Figure 4:**
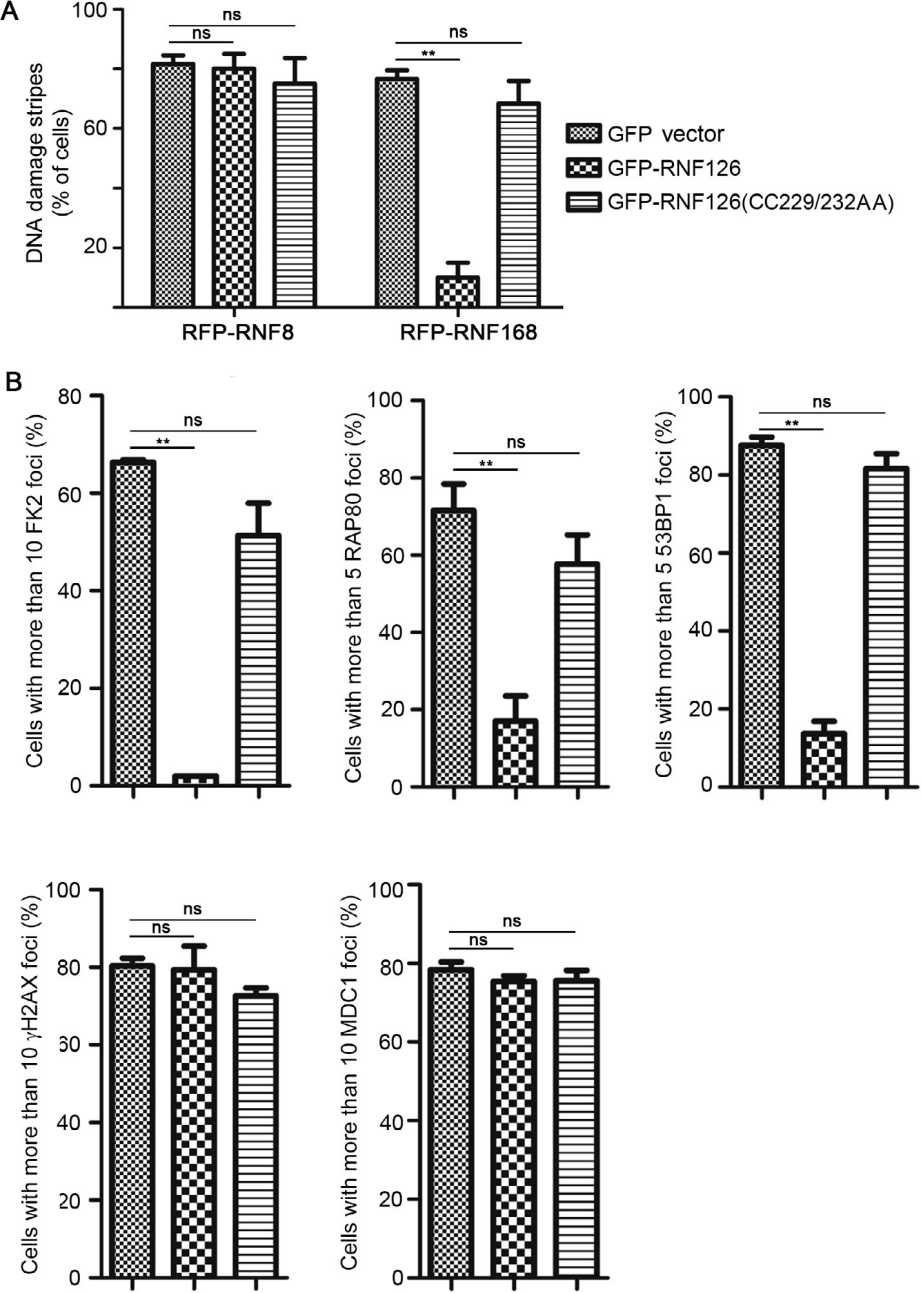
**RNF126 suppresses recruitment of RNF168 and downstream DDR factors to sites of DNA damage** **A.** Over-expression of wild-type RNF126, but not catalytically-inactive RNF168(CC229/232AA), compromises the recruitment of RNF168 but not RNF8 to DNA damage stripes. U2OS cells expressing RFP-RNF8 or RNF168 were transfected with GFP vector, GFP-RNF126, or GFP-RNF126(CC229/232AA) and subjected to UV laser micro-irradiation 48 h after transfection. The percentage of RFP-positive DNA damage stripes compared to cells dually positive for RFP and GFP was quantitated in three randomly-selected fields on the condition that each field had >100 cells dually positive for GFP and RFP. **B.** Over-expression of wild-type RNF126, but not catalytically-inactive RNF168(CC229/232AA), compromises the recruitment of RNF168 downstream factors to bleomycin-induced DNA damage sites. U2OS cells expressing GFP vector, GFP-RNF126 or GFP-RNF126(CC229/232AA) were subjected to bleomycin treatment (10 μg/ml) for 1 h before immunofluorescence staining with the indicated antibodies. The percentage of cells with >5 (for RAP80 and 53BP1) or >10 foci (for FK2, MDC1, and γH2AX) over GFP-positive cells was quantitated in three randomly-selected fields on the condition that each field had >100 GFP-positive cells. Data represent the mean ± SD. A two-way ANOVA was performed. ^**^P < 0.01; ns, not significant; IB, immunoblot; RAP80, receptor-associated protein 80; 53BP1, TP53-binding protein 1; MDC1, mediator of DNA damage checkpoint protein 1; γH2AX, phosphorylated H2A histone family member X.

We continued to explore if overexpression of RNF126 modulates RNF8/RNF168 recruitment to DNA damage site. Surprisingly, over-expression of wild-type GFP-RNF126, but not the catalytically-inactive mutant GFP-RNF126(CC229/232AA), compromised the recruitment of RFP-RNF168 to the micro-irradiation-induced DNA damage stripes (Figure 4A) and the bleomycin-induced focus formation of FK2, RAP80, and 53BP1 (Figure 4B). Over-expression of GFP-RNF126 or GFP-RNF126(CC229/232AA) did not have any impact on the recruitment of RFP-RNF8 to micro-irradiation-induced DNA damage stripes (Figure 4A) or the bleomycin-induced focus formation of MDC1 or H2AX (Figure 4B). These results indicate that RNF126 negatively regulates the recruitment of RNF168 and RNF168-dependent DDR factors to the DNA damage site.

**Figure 5:**
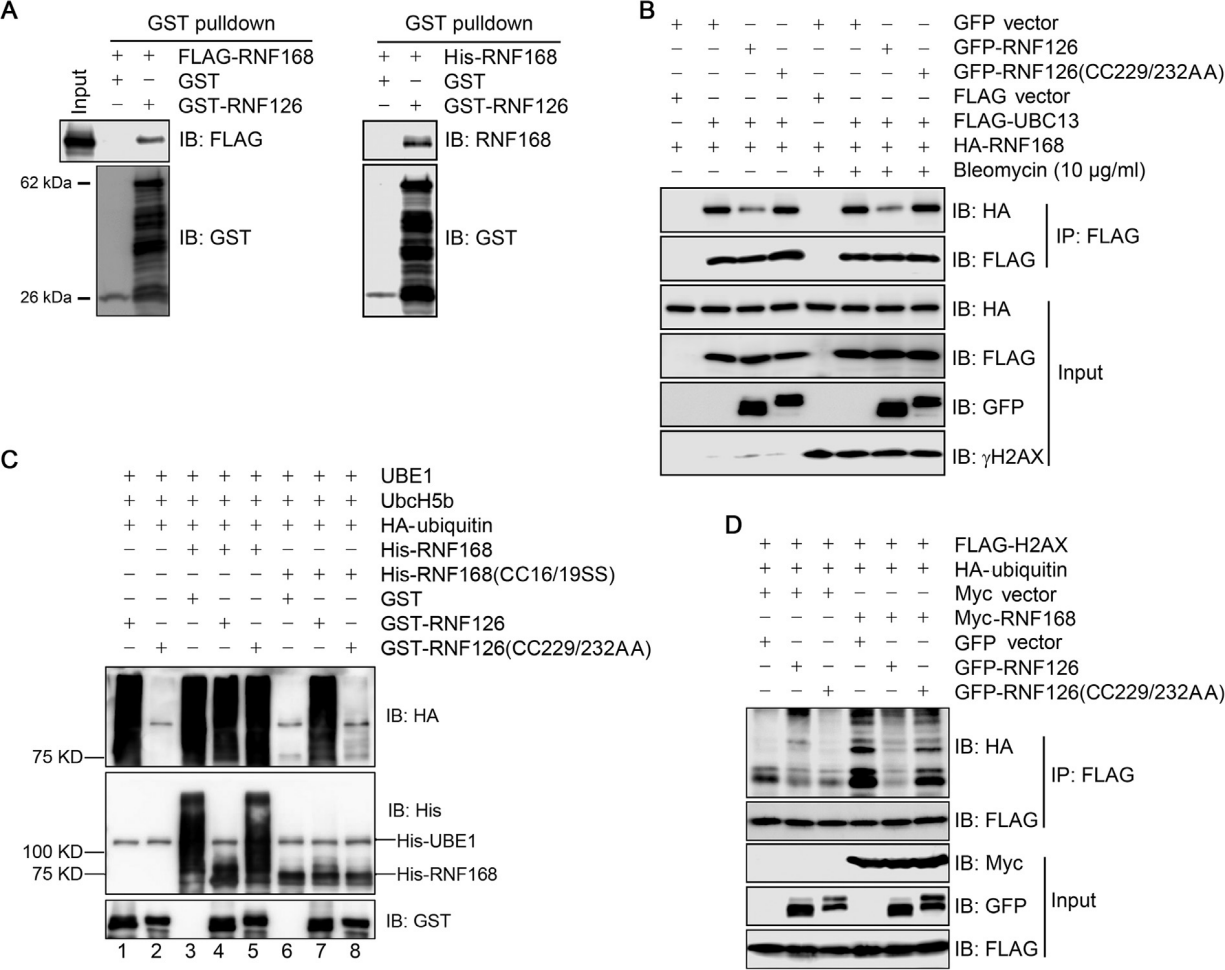
**RNF126 suppresses UBC13 binding to RNF168 and RNF168-mediated H2AX ubiquitination** **A.** RNF126 directly interacts with RNF168. GST pulldown assays were performed using bacterially-produced GST-RNF126 to pull down FLAG-RNF168 present in the total cell lysate of HEK293T cells transiently expressing FLAG-RNF168 or bacterially-produced His-RNF168. **B.** RNF126 negatively modulates the interaction between RNF168 and UBC13. HEK293T cells transiently co-expressing HA-RNF168 and FLAG-UBC13 and GFP-RNF126 or GFP-RNF126(CC229/232AA) were subjected to mock treatment or bleomycin treatment for 1 h. Total cell lysates were extracted with SDS-containing lysis buffer and boiled for 10 min before subjected to immunoprecipitation with anti-FLAG beads followed by immunoblotting with the indicated antibodies. **C.** RNF126 ubiquitinates RNF168 *in vitro. In vitro* ubiquitination assays were performed by incubating bacterially-produced wild-type GST-RNF126 or catalytically-inactive GST-RNF126(CC229/232AA) with wild-type His-RNF168 or catalytically-inactive His-RNF168(CC16/19SS) in the presence of UBE1, UbcH5b, and HA-ubiquitin at 30 °C for 1 h. The reactions were analyzed by immunoblotting with the indicated antibodies. **D.** Over-expression of wild-type RNF126, but not catalytically-inactive RNF126(CC229/232AA), compromises H2AX ubiquitination. Total cell lysates derived from HEK293T cells co-expressing Myc-RNF168 and FLAG-H2AX and GFP-RNF126 or GFP-RNF126(CC229/232AA) were subjected to immunoprecipitation with anti-FLAG beads followed by immunoblotting with the indicated antibodies. IB, immunoblot; IP, immunoprecipitation; UBC13, E2 ubiquitin-conjugating enzyme N; UBE1, ubiquitin-like modifier-activating enzyme 1; UbcH5b, ubiquitin-conjugating enzyme E2 D2.

We further explored how RNF126 modulates RNF168 by *in vitro* pulldown assay. Here, we found that recombinant protein GST-RNF126 (produced in *Escherichia coli*) pulled down FLAG-RNF168 from total cell lysates and recombinant His-RNF168 (produced in *E. coli*) (Figure 5A), indicating that RNF126 directly interacts with RNF168. This finding raised the possibilities that either RNF126 blocks presentation of E2 ubiquitin-conjugating enzyme N (UBC13) to RNF168, or RNF168 is a RNF126 substrate. To test these hypotheses, we co-expressed wild-type GFP-RNF126 or catalytically-inactive GFP-RNF126(CC229/232AA) with FLAG-UBC13 and HA-RNF168. As shown in Figure 5B, wild-type but not mutant GFP-RNF126 reduced the level of HA-RNF168 binding to FLAG-UBC13, and this reduction was independent of bleomycin-induced DNA damage.

Furthermore, *in vitro* ubiquitination assays showed that recombinant wild-type GST-RNF126, but not catalytically-inactive GST-RNF126(CC229/232AA), suppressed RNF168 autoubiquitination (compare lanes 3 and 4 and lanes 4 and 5 in the His blot of Figure 5C) and was able to ubiquitinate the catalytically-inactive His-RNF168(C16/19S) (compare lanes 7 and 8 in the His blot of Figure 5C).

Finally, we examined the impact of RNF126-mediated RNF168 ubiquitination on the immediate RNF168 substrate, H2AX. We co-expressed wild-type GFP-RNF126 or catalytically-inactive mutant GFP-RNF126(CC229/232AA) with myc-RNF168 and FLAG-H2AX in HEK293T cells, immunoprecipitated the anti-FLAG complexes, and purified the proteins under denaturing conditions before immunoblotting to detect ubiquitination levels. Here, over-expression of RNF168 enhanced H2AX ubiquitination, whereas over-expression of wild-type GFP-RNF126, but not catalytically-inactive GFP-RNF126(CC229/232AA) mutant, diminished H2AX ubiquitination (Figure 5D). These results show that RNF126 negatively regulates RNF168-mediated H2AX ubiquitination.

### RNF126 is required for HR repair

**Figure 6:**
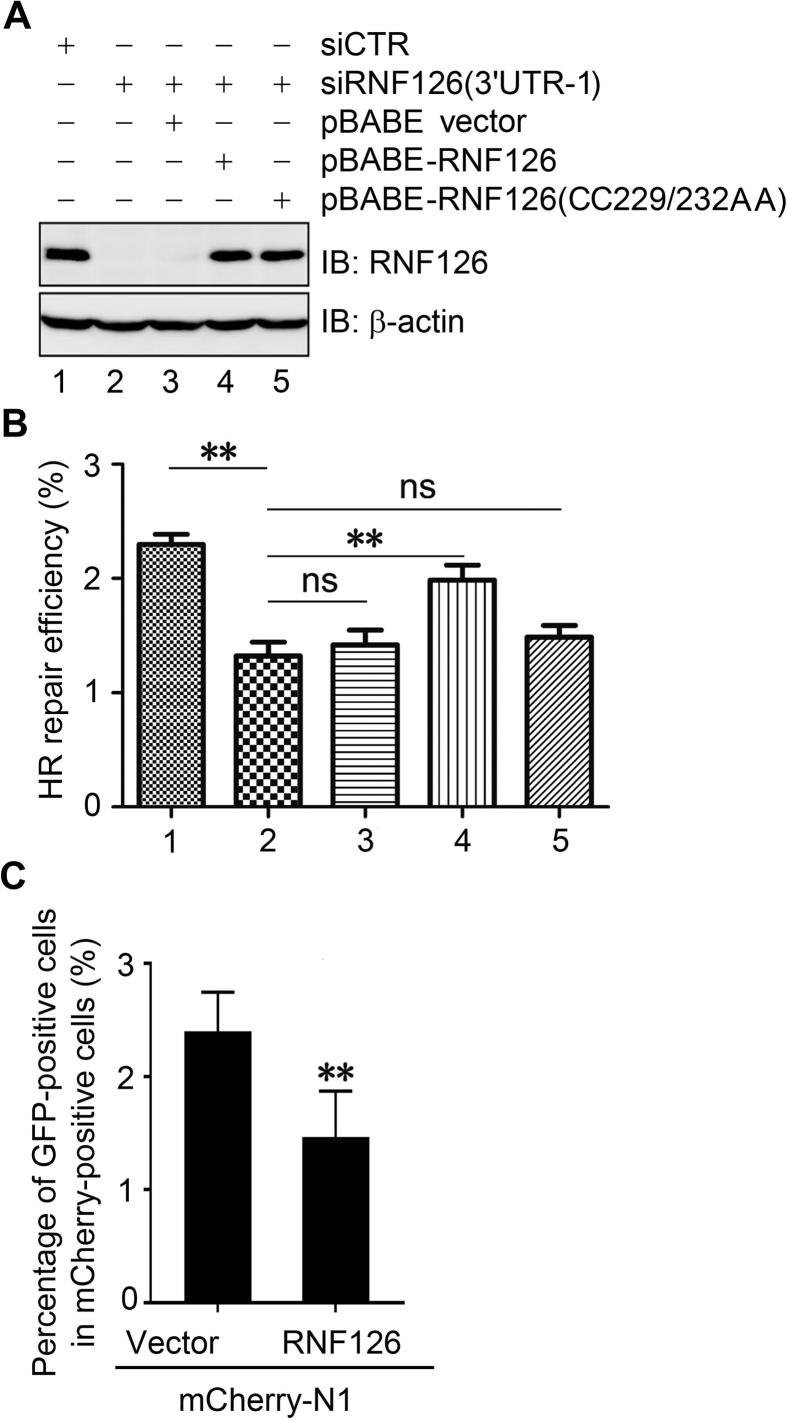
**Maintaining proper levels of RNF126 is essential for HR-mediated DSB repair** **A.** and **B.** Inhibition of RNF126 expression compromises HR-mediated DSB repair. Endogenous expression of RNF126 in DR-U2OS cells was inhibited by transfection with siRNF126 (3′UTR-1) that specifically targeted the 3′UTR of RNF126. Wild-type RNF126 or catalytically-inactive RNF126(CC229/232AA) were re-introduced into RNF126-depleted cells by retroviral infection. A. Expression levels of RNF126 were determined by immunoblotting with an anti-RNF126 antibody. B. HR-mediated DSB repair efficiency was based on the percentage of GFP-positive cells measured by flow cytometry. Three independent experiments were performed. **C.** Over-expression of RNF126 compromises HR-medicated DSB repair. DR-U2OS cells expressing mCherry vector or mCherry-RNF126 was infected with lentiviral particles encoding I-SceI. The percentage of GFP-positive cells over mCherry-positive cells was determined by flow cytometry to indicate the HR repair efficiency. Three independent experiments were performed. Data represent the mean ± SD. A two-way ANOVA was performed. ^**^P < 0.01. IB, immunoblotting; siCTR, siRNA control; HR, homologous recombination; ns, not significant.

To examine the role of RNF126 in HR-mediated DSB repair, we used a GFP-based chromosomal reporter (DR-GFP) in DR-U2OS cells to measure HR efficiency [34]. We found that HR-mediated DSB repair was significantly compromised when RNF126 expression was inhibited by three specific siRNAs targeting the RNF126 3′-UTR. Data from experiments performed using siRNF126 (3′UTR-1) were shown in Figure 6A and B. This siRNF126-mediated impairment of HR was partially rescued by introducing the expression of wild-type pBABE-RNF126, but not the catalytically-inactive pBABE-RNF126(CC229/232AA) mutant (Figure 6A and B). Given that RNF126 negatively modulates RNF168 function in the DSB response, we examined the impact of RNF126 over-expression on HR repair. To this end, mCherry-tagged RNF126 was expressed in DR-U2OS cells, and the ratio of GFP-positive cells in mCherry-positive cells was considered as a readout of the HR repair efficiency in RNF126-expressing cells. As expected, expression of RNF126 compromised HR-mediated DSB repair efficiency (Figure 6C). Taken together, these results imply that maintenance of adequate RNF126 levels is essential for HR-mediated repair.

## Discussion

RNF8 and RNF168 are critical E3 ligases for DSB signaling, and their activity is tightly regulated by additional E3 ligases and mediators. RNF8 does not directly ubiquitinate nucleosomal H2A/H2AX; instead, RNF168, which is recruited to DSB sites in a RNF8-dependent manner, catalyzes ubiquitination of H2A-type histones at K13/15 residues in the N terminus [17]. The molecular cascade underlying RNF8-dependent RNF168 recruitment has been deciphered only recently. Specifically, RNF8 predominantly ubiquitinates linker histone H1 at DSB sites with K63-linkage-type polyubiquitin chains. Ubiquitinated H1 is recognized by RNF168 through its ubiquitin-dependent DSB recruitment module 1 (UDM1) region, which is composed of two ubiquitin-binding motifs (UMI and MIU1) and a flanking target recognition LR motif 1 (LRM1) [17], [18], [23]. In addition, RNF169, independent of its E3 ligase activity, directly recognizes accumulating RNF168-mediated ubiquitination products, including the H2A-ubiquitin mark, and accumulates at DSBs [22], [23], [24]. As such, RNF169 competes with other ligases to bind to RNF168-generated ubiquitination products, thus limiting the magnitude and propagation of the RNF8/RNF168-dependent DSB response.

Our lab and others have recently reported that the scaffold protein BCL10 is recruited to DSBs in an ATM-dependent and RNF8-dependent manner, and the dually phosphorylated and ubiquitinated BCL10 presents UBC13 to RNF168, facilitating RNF168-mediated ubiquitination events [25], [35]. In this report, we identified RNF126 as a novel negative regulator of RNF168. RNF126 negatively regulates RNF168 via one of two possible mechanisms. (1) RNF126 competes with UBC13 to bind to RNF168, reducing the accessibility of RNF168 to its E2; or (2) RNF126 competes with histone H1 to bind to RNF168, as both RNF126 and H1 bind to the same UMI and MIU1 motifs in RNF168 [18]. Our results also support the possibility that direct ubiquitination of RNF168 by RNF126 may have an impact on RNF168 activity. Given that recruitment of RNF126 to the DNA damage site is dependent on both ATM (Figure S1) and RNF8 (Figure 3), the potential phosphorylation of RNF126 by ATM and the potential ubiquitination of RNF126 by RNF8, both of which are being investigated in the lab, may have an impact on the function of RNF126 in the DDR. While preparing this manuscript, Lee et al. demonstrated in an independent study that RNF126 negatively regulates RNF168-mediated events in the DDR [36]. They, however, did not determine the direct molecular mechanisms as to how RNF126 suppresses RNF168. Given that RNF169, RNF126, and BCL10 modulate RNF168-mediated events in the DDR, the functional interplay between these proteins warrants further exploration.

RNF126 protein is ubiquitously expressed in the cytoplasm and nucleus, and has important roles in various intracellular biological processes, dependent or independent of its E3 ligase activity. As an E3 ligase, RNF126 specifically ubiquitinates the mitochondrial protein frataxin (FXN), promoting its degradation [37]. It is of note that reduced FXN expression leads to Friedreich ataxia (FRDA), a severe genetic neurodegenerative disease [38]. This finding suggests that RNF126 may be a promising therapeutic target for FRDA. RNF126 protein is also highly expressed in the invasive breast cancer tissue, where it targets p21 for ubiquitination and subsequent proteasome-mediated degradation, promoting breast cancer cell proliferation [39]. High expression of RNF126 is an independent predictor of a poor prognosis in invasive breast cancer and is considered a potential biomarker for cancer responsiveness to checkpoint kinase 1 (CHK1) inhibitors, as RNF126 increases gene expression of *CHK1* in breast cancer cells [40]. RNF126 also monoubiquitinates the activation-induced cytidine deaminase [33], but the functional consequence of this process remains to be elucidated.

RNF126 is directly involved in DSB signaling and repair via direct Ku80 ubiquitination to release the Ku70/80 complex from the DSB ends, facilitating the completion of NHEJ repair. One study finds that inhibiting RNF126 expression by siRNA reduces NHEJ-mediated repair of I-SceI-induced DSBs [41]. These data, however, are in contradiction to a recent report [36], in which RNF126 overexpression via the same reporter system suppresses NHEJ-mediated DSB repair. Our data show that RNF126 overexpression reduces the number of cells with >5 53BP1 foci induced by bleomycin treatment (Figure 4B) and thus support the conclusions of the latter study. However, the two aforementioned studies [36], [41] could be reconciled as both downregulation and upregulation of RNA126 are found to compromise NHEJ-mediated DSB repair [36], [41], indicating that maintenance of proper levels of RNF126 is essential for NHEJ repair.

As a transcription factor, RNF126 directly interacts with E2F1 to positively promote the transcription of *BRCA1* and thus HR-mediated DSB repair [42]. However, our study demonstrates that restoration of the HR repair defects induced by *RNF126* depletion requires the E3 ligase activity of RNF126 (Figure 6A and B). Furthermore, overexpression of wild-type RNF126 compromises HR repair (Figure 6C). We suspect that the excessive RNF126 could block the accessibility of RNF168 to UBC13 and/or the ubiquitinated linker histone H1.

Our study, along with others, has demonstrated that maintaining proper levels of RNF126 is critical for DSB repair; tilting the balance of RNF126 expression in either direction can lead to a DSB repair defect, genome instability, and ultimately tumorigenesis. Indeed, various cancers listed in The Cancer Genome Atlas reportedly harbor single or combinatorial *RNF126* alterations, including mutations, deletions, or amplifications (Table S1). Therefore, therapeutic strategies targeting RNF126 must consider and be cautious of disturbing the balance of RNF126 expression.

## Materials and methods

### Cell culture

Human osteosarcoma U2OS cells, cervical cancer Hela cells, and SV40 large T antigen-transformed embryonic kidney HEK293T cells were cultured in high-glucose Dulbecco’s Modified Eagle’s Medium (HyClone, Logan, UT, Cat No. SH30022.01) supplemented with 10% fetal bovine serum (PAN-Biotech, Cat No. P30-3302; Aidenbach, Germany) in the presence of 100 U/ml penicillin and 100 µg/ml streptomycin at 37 °C with 5% CO_2_ in a humidified atmosphere.

In addition, DR-GFP U2OS cells that contain one single copy of DR-GFP stably integrated into their genome [34] were also used. The DR-GFP construct contains a tandem repeat of the GFP-coding gene, in which one copy is inactivated by insertion of the sequence recognized by the nuclease I-SceI and the other by truncations at both N- and C-termini. A functional GFP gene can be reconstituted by the expression of I-SceI if the DSB is repaired by HR. The DR-GFP U2OS cells were cultured similarly as described above.

### siRNA transfection

Short interfering RNA (siRNA) oligos were purchased from RiboBio (Guangzhou, China). The details of the oligos were listed in Table S2. Three siRNA oligos for each target gene were tested and data derived from one siRNA oligo for each target gene were shown. siRNA transfections were performed using Lipofectamine RNAiMAX (Cat No.13778-150, Invitrogen, Waltham, MA) according to the manufacturer’s instructions.

### Plasmid construction

Full length RNF126 cDNA was obtained from a cDNA library and cloned into pEGFP-C1 or pcDNA3.0-3HA vectors. The recombinant pcDNA3.0-3Flag-RNF8 and pcDNA3.0-3Flag-RNF168 plasmids were previously generated in our laboratory [25]. Full length RNF126 cDNA was subcloned into the *Bam*HI and *Sal*I sites of pGEX-6P-1. Full length RNF168 cDNA was inserted into the *Sac*Ⅰ and *Xho*Ⅰ sites of pET-28a. Full length RNF168 cDNA was transferred into the *Xho*I and *Apa*I sites of pcDNA3.1-Myc-His. Full length RNF126 cDNA was inserted into the *Bam*HⅠ and *Sal*Ⅰ sites of pBABE-Puro retroviral vector. Full length RNF126, RNF8, and RNF168 cDNAs were subcloned into the *Xho*I and *Eco*RI sites of pLVX-mCherry-N1 vector, respectively. Full length ubiquitin cDNA was inserted into the *Bam*HI and *Apa*I sites of pcDNA3.0-3HA vector. Expression construct of FLAG-UBC3 was described previously [25]. The vectors of pEGFP-C1, pGEX-6P-1, pET-28a, pcDNA3.1-Myc-His, pBABE-Puro, pLVX-mCherry-N1, and pcDNA3.0-3HA were kept in our laboratory. GFP-tagged RNF8, HA-tagged RNF168, and various truncated mutants were constructed using conventional molecular approaches. All constructs were verified by sequencing.

### Antibodies

The following antibodies were used for immunofluorescence and immunoblotting: RNF126 (mouse monoclonal antibody, Cat No. sc-376005, 1:500, Santa-Cruz Biotech, Santa Cruz, CA); FK2 (mouse monoclonal antibody, Cat No. 04-263, 1:250, EMD Millipore, Boston, MA); RNF8 (rabbit polyclonal antibody, Cat No. PABR-1160-042604, 1:500, Bethyl, Montgomery, TX); RNF168 (rabbit polyclonal antibody, Cat No. 06-1130, 1:1000, EMD Millipore); 53BP1 (rabbit polyclonal antibody, Cat No. A300-272A, 1:500, Bethyl); RAP80 (rabbit polyclonal antibody, Cat No. A300-763A, 1:250, Bethyl); γH2AX (mouse monoclonal antibody, Cat No. 05-636, 1:500, EMD Millipore); MDC1 [43]; HA (mouse monoclonal antibody, Cat No. M180-3, 1:5000, MBL, Nagoya, Japan); FLAG (mouse monoclonal antibody, Cat No. M185-3L, 1:5000, MBL); GFP (rabbit polyclonal antibody, Cat No. 598, 1:2000, MBL); β-actin (mouse monoclonal antibody, Cat No.66009-1-Ig, 1:5000, Proteintech, Chicago, IL). The following secondary antibodies were used: donkey anti-mouse IgG (Cat No.715-585-150, 1:1000, Jackson ImmunoResearch, West Grove, PA), donkey anti-rabbit IgG (Cat No. 711-585-152, 1:1000, Jackson ImmunoResearch).

### Immunoprecipitation and immunoblotting

Immunoprecipitation and immunoblotting were performed as previously described [43].

### Immunofluorescence analysis

U2OS cells were transfected with the appropriate constructs for 24 h and then fixed in 4% paraformaldehyde on ice for 10 min. The cells were then permeabilized with 0.1% Triton X-100/PBS on ice for 30 min, and blocked with 5% BSA/PBS plus 0.5% Tween-20 (PBST) at room temperature (RT) for 30 min. The cells were incubated for 1 h with primary antibody at RT, and then washed three times in 0.5% PBST before incubation with the appropriate secondary antibody for 30 min at RT. Images were acquired by confocal microscopy.

### Ubiquitination assays

*In vitro* ubiquitination assays were performed as described previously [37] with slight modifications. In brief, the reaction mixture contained 100 nM recombinant human E1 (His-UBE1, Cat No. E-304, Boston Biochem, Cambridge, MA), 1.4 μM recombinant human E2 (UbcH5b/UBE2D2, Cat No. E2-622, Boston Biochem), and 30 μM recombinant human HA-ubiquitin (Cat No. U-110, Boston Biochem) in ubiquitination buffer [25 mM Tris–HCl (pH 8.0), 100 mM NaCl, 1 mM DTT, 2.5 mM ATP, and 4 mM MgCl_2_]. The recombinant human His-RNF168/His-RNF168(CC16/19SS) and GST-RNF126/GST-RNF126 (CC229/232AA) proteins expressed in *E. coli* were made in the lab and included to a final volume of 50 μl with a final concentration of 1 μM. The reaction mixtures were incubated for 60 min at 30 °C and terminated by adding 5× SDS sample buffer.

### DDR assays

HR-mediated DSB repair reporter assays and UV laser micro-irradiation-induced DNA damage stripes coupled with live-cell imaging analysis were performed as previously described [44].

### Statistical analysis

Quantification data were analyzed by performing a two-way analysis of variance (ANOVA). Differences with *P* < 0.05 were considered significant.

## Authors’ contributions

XX conceived, designed, and supervised the project. LZ performed majority of the experiments with help from ZW, RS, XZ, JZ, and BP. XX and LZ analyzed the data and wrote the paper. All authors read and approved the final manuscript.

## Competing interests

The authors declare that they have no conflicts of interests.
