# Supplementary material for: RNF126 Quenches RNF168 Function in the DNA Damage Response
Source: Genomics Proteomics Bioinformatics. 2018 Dec 4;16(6):428–38. doi: 10.1016/j.gpb.2018.07.004 (PMC6411902; doi:10.1016/j.gpb.2018.07.004)
Supplement: Supplementary Table S1 [file mmc1.rtf]

Table S1  RNF126 genetic alterations in different cancers
Cancer type	No. of cases	Mutation	Amplification	Deletion	
Papillary stomach adenocarcinoma	6	0	0	16.67%	
Prostate neuroendocrine carcinoma	66	0	15.15%	0	
Prostate	65	0	9.23%	0	
Glioblastoma	13	7.69%	0	0	
Tubular stomach adenocarcinoma	39	2.56%	2.56%	2.56%	
Leiomyosarcoma	140	0	5.00%	2.14%	
Mucinous stomach adenocarcinoma	16	0	6.25%	0	
Serous ovarian cancer	318	0	0.31%	5.35%	
Cervical squamous cell carcinoma	167	0.60%	1.20%	3.59%	
Metaplastic breast cancer	19	0	0	5.26%	
Dedifferentiated liposarcoma	101	0	3.96%	0	
Diffuse type stomach adenocarcinoma	51	0	1.96%	1.96%	
Myxofibrosarcoma	28	0	3.57%	0	
Mixed	1000	0	0.90%	2.40%	
Paraganglioma	31	0	3.23%	0	
Note: Data were obtained from www.cbioportal.org in February 2018. Alterations with frequency >3% are included. 
