# Supplementary material for: RNF126 Quenches RNF168 Function in the DNA Damage Response
Source: Genomics Proteomics Bioinformatics. 2018 Dec 4;16(6):428–38. doi: 10.1016/j.gpb.2018.07.004 (PMC6411902; doi:10.1016/j.gpb.2018.07.004)
Supplement: Supplementary Table S2 [file mmc2.docx]

**Table S2 siRNAs used in the current study**

| **Gene** | **siRNA name** | **siRNA sequence** |
| --- | --- | --- |
|  | siCTR | 5’-CGUACGCGGAAUACUUCGAdTdT-3’ |
|  |  |  |
| *RNF126* | siRNF126-A | 5’-GAUUAUAUCUGUCCAAGAUdTdT-3’ |
|  | siRNF126-B | 5’-GCAUCUUCGAUGACAGCUUdTdT-3’ |
|  | siRNF126-C | 5’-GCAGGGCUACGGACAGUUUdTdT-3’ |
|  | siRNF126 (3’UTR-1) | 5’-GUCUAACCUCACCCUCUAAdTdT-3’ |
|  | siRNF126 (3’UTR-2) | 5’-UCACCCUCUAAACGUUCAGdTdT-3’ |
|  | siRNF126 (3’UTR-3) | 5’-GCUUUGAAAUAAACGGACGdTdT-3’ |
|  |  |  |
| *RNF8* | siRNF8(3’UTR-1) | 5’-CCAGCUAUAUGAAGCACUUdTdT-3’ |
|  | siRNF8(3’UTR-2) | 5’-GGAGAAUCCUGGAAUUGUUdTdT-3’ |
|  | siRNF8(3’UTR-3) | 5’-GCUGCUGCUUUACUACAUUdTdT-3’ |
|  |  |  |
| *RNF168* | siRNF168-A | 5’-CTTTAAAGATGCAGTTGAAdTdT-3’ |
|  | siRNF168-B | 5’-GTGGAACTGTGGACGATAAdTdT-3’ |
|  | siRNF168-C | 5’-GGAACTGAGAAGAGAATATdTdT-3’ |
